# Thiolactomide: A New Homocysteine Thiolactone Derivative from *Streptomyces* sp. with Neuroprotective Activity

**DOI:** 10.4014/jmb.2108.08015

**Published:** 2021-09-15

**Authors:** Jun-Pil Jang, Min Cheol Kwon, Toshihiko Nogawa, Shunji Takahashi, Hiroyuki Osada, Jong Seog Ahn, Sung-Kyun Ko, Jae-Hyuk Jang

**Affiliations:** 1 Anticancer Agent Research Center, Korea Research Institute of Bioscience and Biotechnology (KRIBB), Cheongju 28116, Republic of Korea; 2 Department of Biomolecular Science, KRIBB School of Bioscience, University of Science and Technology (UST), Daejeon 34141, Republic of Korea; 3 RIKEN Center for Sustainable Research Science, 2-1 Hirosawa, Wako, Saitama 351-0198, Japan; 4 Natural Products Biosynthesis Research Unit and RIKEN-KRIBB Joint Research Unit, RIKEN Center for Sustainable Research Science, 2-1 Hirosawa, Wako, Saitama 351-0198, Japan

**Keywords:** Thiolactomide, *Streptomyces* sp., *N*-acetyl homocysteine thiolactone, neuroprotective activity, 6-hydroxydopamine

## Abstract

A new homocysteine thiolactone derivative, thiolactomide (1), was isolated along with a known compound, *N*-acetyl homocysteine thiolactone (2), from a culture extract of soil-derived *Streptomyces* sp. RK88-1441. The structures of these compounds were elucidated by detailed NMR and MS spectroscopic analyses with literature study. In addition, biological evaluation studies revealed that compounds **1** and **2** both exert neuroprotective activity against 6-hydroxydopamine (6-OHDA)-mediated neurotoxicity by blocking the generation of hydrogen peroxide in neuroblastoma SH-SY5Y cells.

## Introduction

Natural products isolated from microorganisms serve as useful chemical templates for the development of various lead compounds essential for clinical applications [1, 2]. Recently, various secondary metabolites of actinomycetes have been attracting particular interest, as many of them have unique structural features and interesting biological properties [3, 4] that make them useful as bioprobes, *i.e.*, biochemical tools for investigating cell functions in chemical biology studies [5, 6]. As part of an ongoing research program focused on novel bioactive secondary metabolites of actinomycetes, we recently reported the isolation and structure elucidation of a new benadrostin derivative (RK-144171) and two known compounds, 3-indolylcarbonyl *α*-L-rhamnopyranoside and 2-aminobenzoyl *α*-L-rhamnopyranoside, from a fermentation broth of *Streptomyces* sp. RK88-1441 [7]. Here, we investigated the minor fraction of this fermentation broth and isolated a new homocysteine thiolactone derivative, thiolactomide (**1**), along with a known compound, *N*-acetyl homocysteine thiolactone (**2**). Also in this study, we describe the isolation, structural elucidation, and biological activity of these compounds.

## Materials and Methods

### General Experimental Procedures

The specific rotations were measured on a JASCO P-1020 polarimeter (JASCO Corporation, Japan) that uses a 100 mm glass microcell. UV spectra were recorded on an Optizen 2120 UV spectrophotometer (Mecasys, Korea). The IR spectra were recorded on a Bruker VERTEX80V FT-IR spectrometer (Bruker, Germany). The NMR spectra were recorded on a Bruker Avance HD 800 NMR spectrometer (Bruker) at the Korea Basic Science Institute (KBSI) in Ochang, Korea. Chemical shifts were referenced to a residual solvent signal (DMSO-*d*_6_*δ*_H_ 2.50, *δ*_C_ 39.51). High-resolution electrospray ionization mass spectrometry (HRESIMS) data were acquired with a Q-TOF mass spectrometer (Waters, USA) on a SYNAPT G2. Column chromatography was performed on reversed-phase silica gel (0.075 mm; Cosmosil, Japan). Analytical C_18_ (Cosmosil, 5 μm, 4.6 × 150 mm) and semipreparative C_18_ (Cosmosil, 10 μm, 10 × 250 mm) columns were used for reversed-phase HPLC on a YL900 HPLC system (Young Lin, Korea) equipped with a YL9120 UV/Vis detector (Young Lin) that used HPLC grade solvents (Burdick & Jackson, USA). Open column chromatography was performed with a silica gel (silica gel 60, 0.063-0.200 mm, Merck). Semi preparative C_18_ (Cosmosil 5C_18_-MS-II, 5 μm, 10 × 250 mm) columns were used for HPLC on a YL9100 HPLC system equipped with a photodiode array detector (YL9160) that uses HPLC grade solvents (Burdick & Jackson). Neuroblastoma SH-SY5Y (#CRL-2266) cells were purchased from American Type Culture Collection (ATCC, USA). Finally, 6-OHDA was purchased from Sigma-Aldrich (USA), and a ROS-Glo H_2_O_2_ Assay Kit was purchased from Promega (UK).

### Cultivation and Extraction of the Strain RK88-1441

A BLAST search revealed that the 16S rRNA sequence of the strain RK88-1441 could make it an actinomycete of the genus *Streptomyces*. Therefore, RK88-1441 [8], was cultured in a medium consisting of soluble starch (10 g), yeast extract (1 g), NZ-amine (1 g), and agar (15 g) in 1.0 L of distilled water at pH 7.0. The stock culture was cultured in a 250 ml Erlenmeyer flask containing 50 ml of seed culture medium (soluble starch 1%, yeast extract 0.1%, and tryptone 0.1%) for 3 days at 28°C on a rotary shaker with agitation at 125 rpm. For a large culture (10 L), 1% of the preculture broth was inoculated into 40 × 1,000-ml baffled Erlenmeyer flasks containing 250 ml of modified CDY broth (glucose 2%, soluble starch 1%, meat extract 0.3%, yeast extract 0.25%, K_2_HPO_4_ 0.005%, NaCl 0.05%, CaCO_3_ 0.05%, and MgSO_4_·7H_2_O 0.05%), which were cultured for 8 days at 28°C on a rotary shaker with agitation at 125 rpm. The mixture was then centrifuged, and the supernatant was extracted with EtOAc, while the mycelium was extracted with acetone. After concentrating the residual solvents under reduced pressure, the two portions were combined and dried to yield 2.2 g of the *Streptomyces* sp. RK88-1441 extract.

### Isolation of Compounds **1** and **2**

The dried extract (2.2 g) was separated by silica gel column chromatography (CHCl3/MeOH, gradient 50:1–0:1 (v/v)) into seven fractions. Fraction 2 was further purified by HPLC with isocratic elution using 20% aqueous MeOH to yield compounds **1** (1.8mg) and **2** (12mg).

*Thiolactomide* (**1**): white amorphous powder; [α]_D_^26^–16 (c 0.1, MeOH); UV (MeOH) *λ*_max_ (log e) 233 (3.37); IR (ATR) *ν*_max_ (cm^-1^) 3272, 1779, 1700, 1648, 1137, 1056; ^1^H and ^13^C NMR data, Table 1; HRESIMS *m/z* 188.0749 [M + H]^+^ (calcd for C8H14NO2S, 188.0745).

**N*-acetyl homocysteine thiolactone (**2**):* white needles; [α]_D_^26^–21 (c 0.1, MeOH); HRESIMS *m/z* 160.0420 [M + H]^+^ (calcd for C_6_H_10_NO_2_S, 160.0420).

### Cell Culture

SH-SY5Y cells were cultured in Dulbecco’s modified Eagle’s medium (DMEM) (Welgene, Korea) supplemented with 10% fetal bovine serum (Welgene) and 1% penicillin/streptomycin (Gibco, USA) in a humidified incubator at 37°C with 5% CO_2_.

### Cell Viability Assay

The proliferation of the SH-SY5Y cells was estimated using the WST colorimetric assay. Briefly, SH-SY5Y cells were seeded at a density of 1 × 10^4^ in 96-well plates and treated with various concentrations of 6-OHDA (0-30 μM), and compounds **1** and **2** (0-10 mM) for 48 h. The wells were then treated with various concentrations of **1** and **2** in the presence or absence of 6-OHDA for 48 h. After incubation, 10 μl of EZ-Cytox solution (DoGen, Korea) was added to each well, followed by incubation for 2 h. The treated cells were then measured at 450 nm using a microplate spectrophotometer (SpectraMax 190, Molecular Devices, USA). *N*-acetylcysteine (NAC) (Sigma-Aldrich, USA) was used as a positive control (5 mM).

### Cellular H_2_O_2_ Generation Assay

The generation of H_2_O_2_ in SH-SY5Y cells was measured using the ROS-Glo H_2_O_2_ assay (Promega, UK). Briefly, SH-SY5Y cells were seeded at a density of 1 × 10^4^ in 96-well plates and incubated overnight. The wells were then treated with various concentrations of **1** and **2** in the presence or absence of 6-OHDA for 48 h, and then ROS-Glo H_2_O_2_ detection substrate was added to the test wells for 20 min. The samples were measured using a luminescence plate reader (Victor X2, Perkin Elmer, USA). ROS-Glo H_2_O_2_ detection solution was prepared according to the manufacturer’s protocol.

### Statistical Analysis

All data are presented as mean ± standard error of the mean (SEM) of at least three independent results. The average and relative SEM were calculated on GraphPad Prism, version 8.4.3 (GraphPad Software, USA). Differences less than 0.05 (*p* < 0.05) were statistically significant.

## Results

### Structural Determination of Compounds

**Fig. 1:**
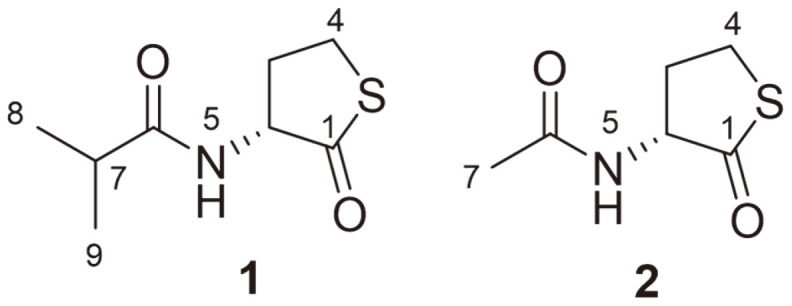
Chemical structures of compounds **1** and **2**.

**Fig. 2:**
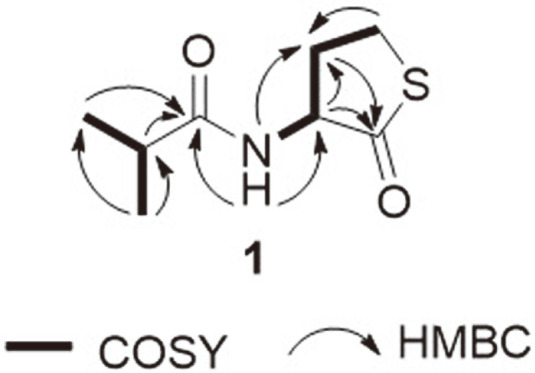
Key COSY and HMBC correlations of compound **1**.

Compound **1** was isolated as a white amorphous powder and its molecular formula was established as C_8_H_13_NO_2_S based on HR-ESI-MS analysis. The mass of **1** was 28 amu (*e.g.*, two CH3 moieties) higher than that of compound **2**, while its planar structure was similar to that of compound **1** based on their ^1^H and ^13^C NMR spectra (Table 1). In particular, only the signals of the methyl [H_3_-8 (*δ*_H_ 1.03) and H_3_-9 (*δ*_H_ 1.01)] and methine [H-7 (*δ*_H_ 2.38)] groups and the lack of a methylene proton were different from the spectroscopic data of **2**. The structure of **1** was further clarified by 2D-NMR spectroscopy (COSY, HSQC, and HMBC) (Fig. 2). The COSY spectrum revealed that compound **1** consists of two partial structures. In the first partial structure, the exchangeable NH proton (*δ*_H_ 8.07) correlated with the methine proton H-2 (*δ*_H_ 4.59), which also correlated with H_2_-3 (*δ*_H_ 2.40 and 2.07). Moreover, the protons at position 3 correlated with H_2_-4 (*δ*_H_ 3.39 and 3.28). In the second partial structure, the methine proton H-7 (*δ*_H_ 2.38) correlated with the two methyl groups (H3-8, *δ*_H_ 1.03 and H_3_-9, *δ*_H_ 1.01). The two structures were further confirmed by HMBC, where H_3_-8 and H_3_-9 correlated with the methine carbon C-7 (*δ*C 34.4) and the amide carbonyl carbon C-6 (*δ*C 176.5), and H-2 (*δ*_H_ 4.59) and H_2_-3 (*δ*_H_ 2.40 and 2.07) correlated with the carbonyl carbon C-1 (*δ*C 205.8). In addition, the HMBC correlations of the NH proton (*δ*_H_ 8.07) to C-2 (*δ*C 58.4) and C-6 (*δ*C 176.5) confirmed the connectivity of the two partial structures. Based on these data, we concluded that the structure of **1** was that of an isobutyryl homocysteine thiolactone (Fig. 1).

Furthermore, compound **1** displayed a negative optical rotation ([α]_D_^26^–16), similar to compound **2**, indicating that the absolute configuration at C-2 was **R**. Therefore, compound **1** was designated as thiolactomide. Compound **2** was identified as *N*-acetyl homocysteine thiolactone through comparison with previously reported data [9, 10].

### Biological Evaluation

**Fig. 3:**
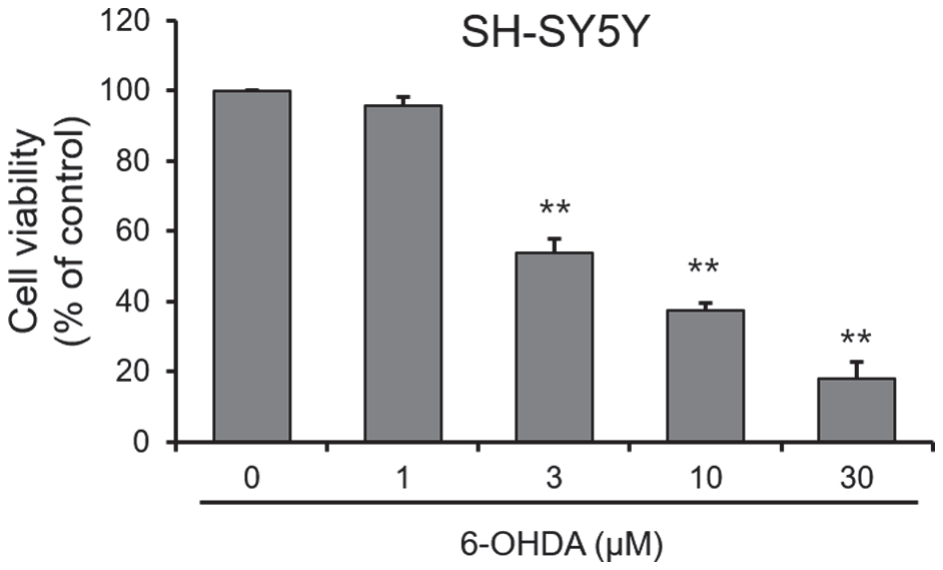
Cytotoxic effects of 6-OHDA in SH-SY5Y cells. Cells were treated with the indicated concentration of 6-OHDA for 48 h. Cell viability was determined by the EZ-Cytox assay. *p < 0.05 and **p < 0.01 vs. control (DMSO: 0).

**Fig. 4:**
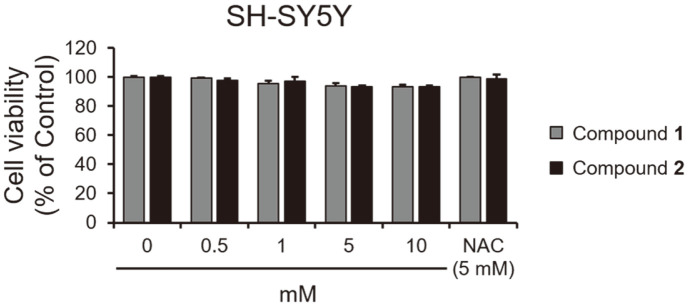
Cytotoxic effects of compounds **1** and **2** in SH-SY5Y cells. Cells were treated with the indicated concentration of compounds **1** and **2** for 48 h. Cell viability was determined by the EZ-Cytox assay. *p < 0.05 and **p < 0.01 vs. control (DMSO: 0). NAC was used as a positive control.

**Fig. 5:**
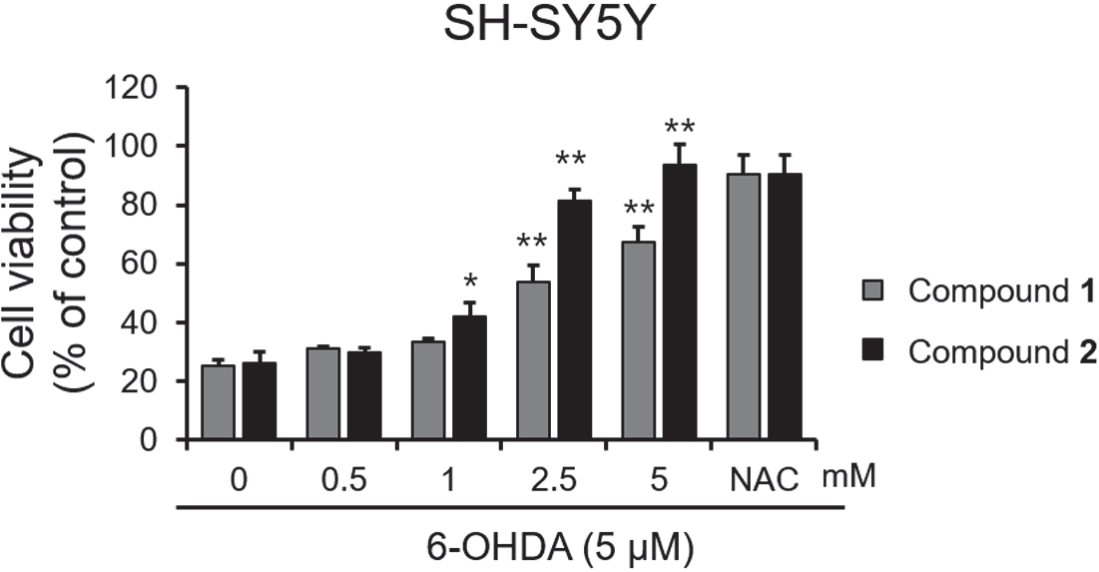
Protective effects of compounds **1** and **2** against 6-OHDA-induced neurotoxicity in SH-SY5Y cells. Cells were treated with 6-OHDA (5 μM) and the indicated concentration of compounds **1** and **2** for 48 h. Cell viability was determined by the EZ-Cytox assay. NAC was used as a positive control. *p< 0.05 and **p < 0.01 vs. control (DMSO: 0).

Neuroexcitotoxicity and oxidative stress play a significant role in neurodegenerative disorders such as Alzheimer’s disease, ischemic stroke, and Parkinson’s disease [11-14]. The neurotoxic agent 6-hydroxydopamine (6-OHDA) is known to induce dementia and brain injury by generating ROS [15, 16]. In our results, 6-OHDA exhibited cytotoxicity to SH-SY5Y neuroblastoma cells at above 3 μM (Fig. 3). Furthermore, the cell viability tests showed that compounds **1** and **2** were non-toxic to SH-SY5Y cells (Fig. 4). Both exhibited potent neuroprotective activity in SH-SY5Y neuroblastoma cells with EC_50_ values of 2.96 ± 0.7 and 1.71 ± 0.32 mM, respectively, as both compounds inhibited 6-OHDA-induced neurotoxicity (Fig. 5).

**Fig. 6:**
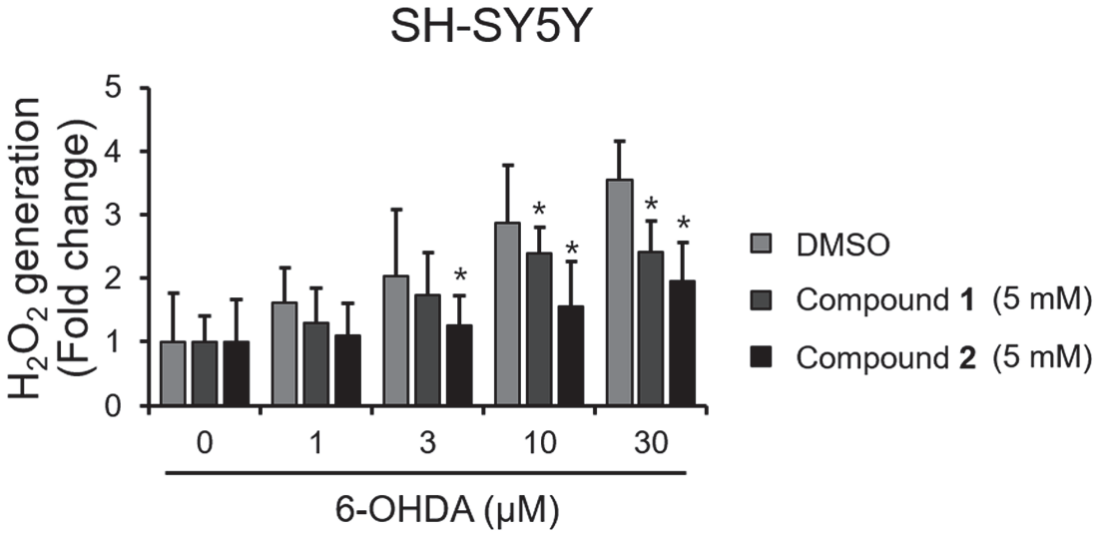
Inhibitory effects of compounds **1** and **2** against 6-OHDA-induced H_2_O_2_ generation in SH-SY5Y cells. Cells were treated with indicated concentration of 6-OHDA and 5 mM of compounds **1** and **2** for 48 h. Intracellular H_2_O_2_ levels were measured using the ROS-Glo H_2_O_2_ Assay Kit. *p< 0.05 vs. control (DMSO: 0).

To further confirm the mechanism of action of the isolated compounds, we investigated the level of 6-OHDA-mediated H_2_O_2_ generation in the presence or absence of **1** or **2**. As shown in Fig. 6, compounds **1** and **2** effectively blocked the level of H_2_O_2_ induced by 6-OHDA, but treatment with 6-OHDA alone favored the generation of H_2_O_2_ in a dose-dependent manner.

## Discussion

We isolated a new homocysteine thiolactone derivative, thiolactomide (**1**), and a known compound, *N*-acetyl homocysteine thiolactone (**2**), from the minor fraction of a fermentation broth of *Streptomyces* sp. RK88-1441.

*N*-acetyl homocysteine thiolactone has been found to perform many beneficial physiological roles [17, 18], as well as having several interesting pharmaceutical applications. For example, *N*-acetyl homocysteine thiolactone has been used as a mucolytic and muco-regulating drug [19]. It is also frequently used for the treatment of chronic hepatitis [20].

In the present study, we evaluated the neuroprotective effects of isolated compounds **1** and **2** against 6-OHDA-mediated ROS stress. Both analogues were non-toxic toward neuroblastoma SH-SY5Y cells and exhibited neuroprotective activities with EC_50_ values of 3.96 ± 0.7 and 1.71 ± 0.32 mM, respectively. Further tests also showed that both compounds **1** and **2** can effectively reduce the generation of H_2_O_2_ induced by 6-OHDA, indicating that they may serve as neuroprotective agents for the treatment of Parkinson’s and Alzheimer’s diseases.

## Figures and Tables

**Table 1:** NMR data for thiolactomide (1) in DMSO-*d*_6_

Position	Thiolactomide (1)	

	δ_C_	δ_H_ (*J* in Hz)
1	205.8	
2	58.4	4.59 (m)
3	30.6	*a* 2.07 (m)
		*b* 2.40 (m)
4	27.1	*a* 3.28 (m)
		*b* 3.39 (m)
5-NH		8.07 (d, 8.3)
6	176.5	
7	34.4	2.38 (quint, 7.0)
8	20.0	1.03 (3H, d, 7.0)
9	19.8	1.01 (3H, d, 7.0)

^1^H and ^13^C data were recorded at 800 and 200 MHz, respectively.
